# Accuracy of Devereux and Teichholz formulas for left ventricular mass calculation in different geometric patterns: comparison with cardiac magnetic resonance imaging

**DOI:** 10.1038/s41598-023-41020-9

**Published:** 2023-08-28

**Authors:** Krunoslav Michael Sveric, Barış Cansız, Anna Winkler, Stefan Ulbrich, Georg Ende, Felix Heidrich, Michael Kaliske, Axel Linke, Stefanie Jellinghaus

**Affiliations:** 1 Department for Internal Medicine and Cardiology, Herzzentrum Dresden, Technische Universität Dresden, Fetscherstr. 76, 01307 Dresden, Germany; 2 Institute for Structural Analysis, Technische Universität Dresden, 01062 Dresden, Germany

**Keywords:** Cardiology, Echocardiography

## Abstract

Left ventricular (LV) myocardial mass is important in the evaluation of cardiac remodeling and requires accurate assessment when performed on linear measurements in two-dimensional echocardiography (Echo). We aimed to compare the accuracy of the Devereux formula (DEV) and the Teichholz formula (TEICH) in calculating LV myocardial mass in Echo using cardiac magnetic resonance (CMR) as the reference method. Based on preceding mathematical calculations, we identified primarily LV size rather than wall thickness as the main source of bias between DEV and TEICH in a retrospective derivation cohort (n = 1276). Although LV mass from DEV and TEICH were correlated with CMR, TEICH did not show a proportional bias as did DEV (− 2 g/m^2^ vs. + 22 g/m^2^). This could be validated in an independent prospective cohort (n = 226) with symptomatic non-ischemic heart failure. DEV systematically overestimated LV mass in all tiers of LV remodeling as compared to TEICH. In conclusion, the TEICH method accounts for the changes in LV geometry with increasing LV mass and thus better reflects the different pattern of LV remodeling than the DEV method. This has important clinical implications, as TEICH may be more appropriate for use in clinical practice, rather than DEV, currently recommended.

## Introduction

**Figure 1:**
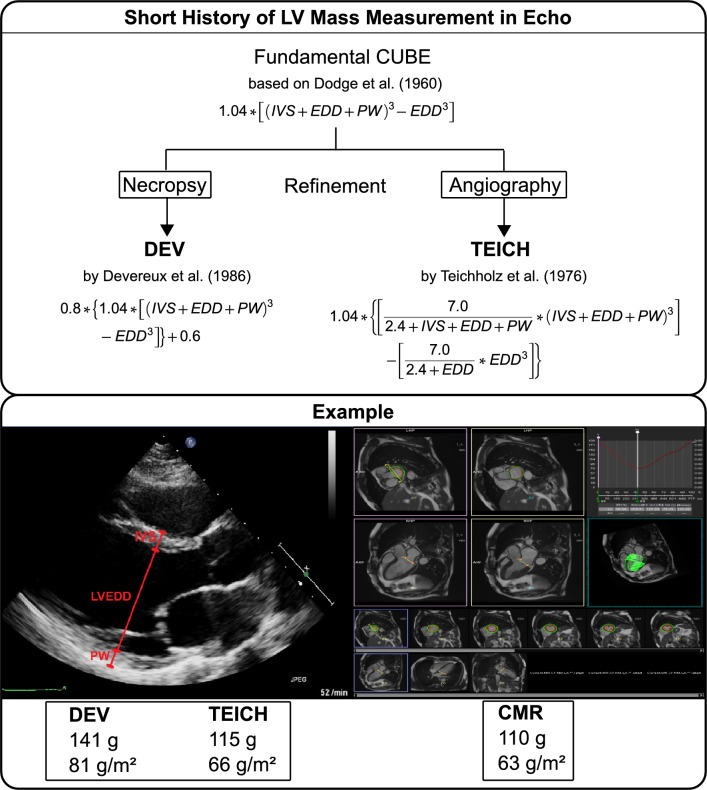
Short historical overview of the LV mass calculation (upper panel). Exemplary measurement of LV EDD, IVS and PW in the parasternal long axis of an Echo. The formula and corresponding myocardial mass calculations for the DEV and TEICH method as well as the results from CMR imaging are shown below, for the same person (lower panel).

Assessing left ventricular (LV) myocardial mass is important in evaluating a patient’s cardiovascular risk and prognosis^1–3^. Though cardiac magnetic resonance (CMR) imaging is regarded as the gold standard for non-invasive measurement of LV mass and morphology, transthoracic echocardiography (Echo) remains the daily armamentarium of cardiologists, due to availability and feasibility^4^. The current Echo guidelines recommend using the DEV method, based on Devereux’s formula, for LV myocardial mass assessment with one-dimensional linear measurements^5,6^. This method is recommended due to its simplicity and reproducibility for screening in large groups. Recently, doubts have arisen about the usefulness of the DEV method as it overestimates LV mass compared to CMR imaging in LVs with distorted morphology^7–9^. An alternative linear measurement method, the TEICH method, based on the Teichholz formula, has been validated by angiography and uses the same measurements as the DEV method^6,10^. As depicted in Fig. 1, both the DEV and TEICH methods rely on the assumption that the LV is a prolate ellipsoid based on the cubic (CUBE) equation developed and refined by Dodge et al.^11,12^. Indeed, alternative measurement methods with higher agreement and precision have been proposed for Echo^13,14^, but require multiple measurements or depend on image quality, such as in three-dimensional Echo. However, the TEICH method has not been systematically evaluated on a large cohort of patients for its feasibility and accuracy. Thus, our study aims to address this gap in knowledge by testing the accuracy of the linear TEICH method. Specifically, we plan to evaluate the DEV and TEICH equations mathematically to understand the impact of measurement parameters on LV mass calculation. Using CMR as the reference, we will identify confounders that cause differences between the two methods and validate our findings in an independent cohort of heart failure patients with different types of LV geometry. Our approach offers a novel and systematic way to assess the differences between these methods. Furthermore, it will provide insights into their practicality in terms of accuracy as compared to the current Echo method.

## Methods

The study was approved by the ethics committee of the Technische Universität Dresden, Dresden, Germany, (Reference number: 157052018), and all patients included prospectively in the validation cohort (see below) provided written informed consent. For patients included retrospectively for the derivation cohort (see below), written informed consent was waived by the ethics committee of the Technische Universität Dresden, Dresden, Germany, (Reference number: 284092012). The study was performed in accordance with the Declaration of Helsinki of 1964 and later revisions.

### Study population

Two timely independent cohorts were analyzed in this study:

For the derivation cohort we screened 4309 consecutive patients who were referred for CMR imaging to our institution for further diagnostic work-up from January 2016 to December 2019, and met the following inclusion criteria: (1) CMR and Echo examinations were performed within 48 h, (2) Echo imaging conformed to current Echo guidelines^5^ and (3) CMR image acquisition followed the standard in-house protocol as described below. Among them (n = 1710), we excluded patients with unsatisfactory image quality in Echo (n = 298) and unsatisfactory CMR protocol adherence or quality (n = 136). Finally, a total of 1276 patients were included. A flow chart of the screening procedure is provided in the Supplemental data (Fig. S1).

The validation cohort (n = 226) was obtained from an ongoing prospective observational study, the Myocardial Heart Failure Study, conducted at our institution since December 2019. Patients with symptomatic heart failure other than ischemic or valvular heart disease underwent standardized Echo and CMR imaging within 24 h before a myocardial biopsy. The primary aim for the study and for myocardial biopsy is to characterize molecular and cellular changes in the myocardium of patients with heart failure, other than ischemic or valvular heart disease. The Echo examinations conformed to current guidelines^5^ and were performed by two experienced cardiologist. CMR acquisition followed our in-house standard protocol. All patients provided written informed consent.

### Echocardiographic imaging and analysis

All Echo images were obtained using a commercial ultrasound imaging system (EPIQ CV or IE33, Philips Healthcare, Andover, Massachusetts, USA). B-Mode directed measurements of LV end-diastolic diameter (EDD) and intraventricular septum (IVS) and posterior wall (PW) at end-diastole from the LV parasternal long-axis view were manually reassessed as depicted in Fig. 1. Care was taken to measure wall thickness and diameters perpendicular to the LV long-axis approximately at the level of the mitral valve leaflet tips^5^*.*

### Calculation of left ventricular myocardial mass in echocardiography

LV myocardial mass was calculated offline from the Echo measurements using following equations:

(i) DEV method applied the Devereux formula^6^ :

(ii) TEICH method used the Teichholz formula^10^ :

$$ \left( i \right)LV\;Mass\left( g \right) = 0.8*1.04*\left[ {\left( {\left[ {EDD + IVS + PW} \right]^{3} - EDD^{3} } \right)} \right] + 0.6\;\left( {{\text{DEV}}} \right) $$

$$ \begin{aligned} \left( {ii} \right)LV\;Mass\left( g \right) = & 1.04* \left\{ {\left[ {7.0/(2.4 + {\text{ IVS + EDD + PW}})} \right]*({\text{IVS + EDD + PW}})^3} \right. \\ & \left. { - [7.0/(2.4 + {\text{EDD}})]{\text{*EDD}}^3} \right\}\;\left( {{\text{TEICH}}} \right) \\ \end{aligned} $$

where EDD, IVS, and PW assume dimensions in centimeters.

### Cardiac magnetic resonance imaging and analysis

According to standardized protocols^15^, CMR images were obtained using a 1.5-Tesla scanner (MAGNETOM Aera, Siemens Healthcare, Erlangen, Germany) (18-channel body array and 32-channel spine array coil). Balanced steady-state free precession electrocardiogram-triggered cine images were acquired using retrospective gating and parallel imaging techniques during 10- to 15-s breath-holds with a temporal resolution of 20–30 frames per cardiac cycle. 4-chamber, 2-chamber, 3-chamber, and short-axis views with a slice thickness of 6 mm with 1.6-mm gaps were acquired in all patients. In addition, late gadolinium enhancement (LGE) imaging was performed 7–10 min after the administration of 0.15 mmol/kg of intravenous gadolinium chelate contrast agent (Dotarem, Guerbet, Sulzbach, Germany) applying delayed enhancement images of segmented inversion-recovery spoiled echo gradient sequences (2D LGE: 1.3 mm × 1.3 mm × 8 mm). CMR images were analyzed using a dedicated post-processing software (Syngo.Via Software VB 70, Siemens, Erlangen, Germany) as shown in Fig. 1. The LV endocardial and epicardial boundaries were traced automatically in short-axis slices at end-diastole and end-systole, using the blood pool method. This process excluded papillary muscles in mass calculation and included these in the LV volume measurements. By incorporating long-axis views, the position and orientation of the mitral valve plane could be established. Consequently, volumetric contour measurements were limited to the ventricular section extending up to the mitral valve plane. LV mass was determined by volumetric calculations of the end-diastolic endocardial and epicardial mesh of the myocardium without papillary muscles and applying a constant of 1.05 for myocardial density. The results of all tracings were supervised and adjusted when necessary and the presence of LV myocardial LGE was identified visually by cardiologists experienced in CMR imaging (> 4 years).

### Tier classification of left ventricular geometry

We stratified the patient population according to the presence/absence of LV hypertrophy and/or LV end-diastolic dilation into four tiers of LV geometry. In brief, tier 1 represents normal LV end-diastolic volumes without LV hypertrophy, tier 2 is characterized by LV dilation without LV hypertrophy, tier 3 comprises no LV dilation but LV hypertrophy, and tier 4 exhibits both LV dilation and LV hypertrophy. Using previously published CMR reference values^16^, categories of LV hypertrophy and LV end-diastolic dilation were defined a priori relative to sex-matched subjects with > 2 standard deviation (SD) above their respective mean reference values : For females: LV mass index > 59 g/m^2^ and LV end-diastolic volume index > 93 ml/m^2^; For males: LV mass index > 75 g/m^2^ and LV end-diastolic volume index > 107 ml/m^2^ (see Table S1 in the Supplemental data).

### Mathematical calculations preceding the study

The mathematical calculation of LV myocardial mass was performed using the (i) DEV and (ii) TEICH equation for Echo as described^6,10^. First, for each equation EDD was incrementally increased, ranging from very small 1 cm to very dilated 7 cm, while mean wall thickness (i.e. mean of IVS and PW) was kept constant for the following levels: 0.8, 1.0, 1.2 and 1,5 cm. The mean wall thickness was incrementally increased, ranging from very thin 0.5 cm to very thickened 1.8 cm, while EDD was kept constant for the following levels: 2.5, 4.5, 5.0 and 6.5 cm. The expanded ranges and levels of the variables used in this calculations are based on previous reports for a wide range of etiologies of cardiac diseases^7,17,18^. Second, based on these univariable calculations, we performed bivariable calculations with concomitant continuous changes in EDD and mean wall thickness. The differences of LV mass results between DEV and TEICH were plotted as contour plots with the corresponding EDD and mean wall thickness. The modeling was used to show the specific output characteristics in LV mass calculation for the DEV or TEICH equations, and to reveal discrepancies based on the different LV geometric assumptions and calculation methods.

#### Observer variability

Intra- and inter-observer variability for LV CMR analysis of mass and end-diastolic volume, as well as for Echo measurements of EDD, IVS, and PW used in mass calculations with the TEICH and DEV formulae, were evaluated in 30 randomly chosen patients.

### Statistical analysis

All statistical analyses were performed with R, the open-source software (version 3.0.2, 2013, The R Foundation for Statistical Computing, Vienna, Austria). Statistical significance was defined as a two-tailed *P* value < 0.05. Sample size calculations (*p* = 0.05; power = 0.90) using data from previous studies^7,9,19,20^ on repeated measurements for agreement between methods^21^ (mean difference = 19 g/m^2^; mean standard deviation = 18 g/m^2^; mean agreement interval = 63 g/m^2^) and for the tier analysis (groups = 4; effect size = 0.2) resulted in n = 100 of repeated measurements/observations. Continuous variables are expressed as mean ± standard deviation (SD) or median with interquartile range (25th quartile; 75th quartile) as appropriate, and compared using a paired/unpaired Student’s t-test or Wilcoxon test if not normally distributed (i.e., via Shapiro–Wilk test). In multiple pairwise comparisons the Bonferroni correction was applied. Categorical variables were reported as counts (%), and compared using a Chi-square or Fisher’s exact test as appropriate. Inter-technique comparisons of parameters of LV mass with intraclass correlation coefficient (ICC), simple linear regression equation and Bland–Altman analysis were applied to assess the bias and limits of agreement (± 1.96*SD). In addition, we assessed intra- and inter-observer agreement using ICC with one- and two-way random effects models for absolute agreement, and determined inter-observer variability using the coefficient of variation (CV)^22^. For comparison purposes, LV mass was related to body surface area. To evaluate the measurement bias between TEICH and DEV as well between Echo and CMR in the derivation cohort, multivariable least square regression analyses were carried out separately, respectively, with following parameters: LV EDD, mean wall thickness (average of PW and IVS due to assumed collinearity), any presence of LGE, sex, and age. Continuous variables were fitted using restricted cubic splines (4 knots) so as to not assume linear relationships between them and the response variable^23^. Hereby, regression results of bias were presented as inter-quartile range effects and depicted as adjusted marginal effects. The multivariable regression assumptions and diagnostics of the models were visually inspected. The coefficient of determination of the multivariable regression model is given as adjusted R^2^ and as predictive R^2^. The predictive R^2^ is based on repeated tenfold cross-validation measuring the goodness of fit and therefore represents an internal validation of the presented model^24^.

## Results

### Mathematical calculation preceding the study

**Figure 2:**
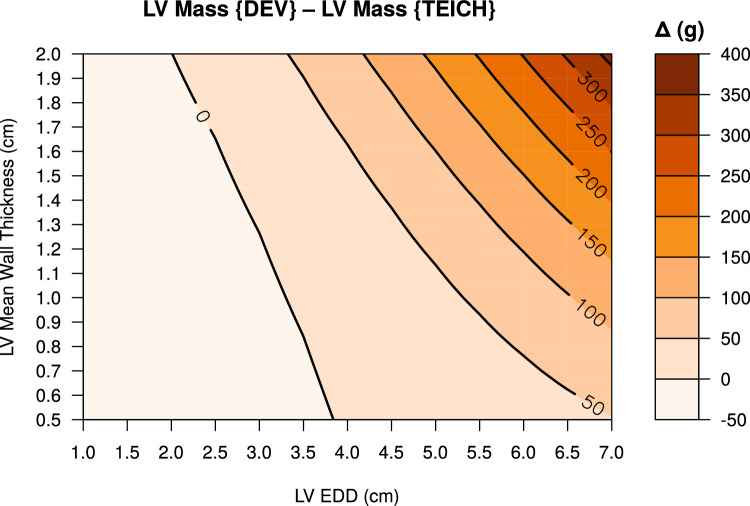
The contour plot represents the differences (Δ) of modeled LV mass between the DEV and TEICH equation in grams (g), elaborating the systematic overestimation of the DEV equation. Mathematical calculations are based on incremental continuous increase of LV EDD and of mean wall thickness [(IVS + PW)/2].

Mathematical calculations showed that the DEV method disproportionately increased calculated mass, especially as a function of LV EDD, compared to the TEICH method (Fig. 2). These differences intensified with increasing mean wall thickness. In contrast, both methods showed good agreement for left ventricular end-diastolic diameters ≤ 4.5 cm and mean wall thicknesses ≤ 1.0 cm. Univariable mathematical calculations for the DEV and TEICH methods are presented in the Supplemental data (Fig. S2).

### Characteristics of the studied cohorts

The anthropomorphic and clinical characteristics of the derivation (n = 1276) and validation (n = 226) cohort are summarized in Table 1. Both cohorts had similar median ages (64 and 62 years, respectively) and a predominance of male patients. Left ventricular ejection fractions ranged from 15 to 85%. In the derivation cohort, the most common indication for CMR and Echo was suspected or progressive coronary artery disease (59%) and suspected or known myocarditis (19%), while suspected hypertrophic cardiomyopathy accounted for only 10%. In contrast, the validation cohort excluded coronary or valvular heart disease according to the study protocol and included more cases of suspected hypertrophic cardiomyopathy (36%), non-ischemic dilated cardiomyopathy (35%), and suspected or chronic myocarditis (26%).

**Table 1:** Main baseline characteristics of the derivation and validation cohort.

	Derivation cohort (n = 1276)	Validation cohort (n = 226)
Age, years	64 (51;75)	61 (53;72)
Males, n (%)	828 (65)	156 (69)
Height, cm	174 (168;179)	176 (167;183)
Weight, kg	81 (73;91)	80 (71;90)
Body surface area, m^2^	1.98 (1.85;2.14)	2.01 (1.84;2.15)
Body mass index, kg/m^2^	26.9 (24.6;29.7)	25.9 (23.8;29.5)
Indication for ECHO and CMR		
Coronary artery disease (existing or suspected), n (%)	755 (59)	0 (0)
Myocarditis (acute or suspected), n (%)	244 (19)	59 (26)
Hypertrophic cardiomyopathy (existing or suspected), n (%)	131 (10)	80 (36)
Non-ischemic dilated cardiomyopathy, n (%)	83 (7)	86 (35)
Other, n (%)	70 (5)	1 (0)
Left ventricular ejection fraction, % units	56 (43;63)	46 (25;52)

Data are presented as median with interquartile ranges (25th percentile; 75% percentile) or as absolute numbers with percentage (%).

CMR, cardiac magnetic resonance; ECHO, echocardiography.

### Inter-method comparisons of measurements of left ventricular myocardial mass

**Figure 3:**
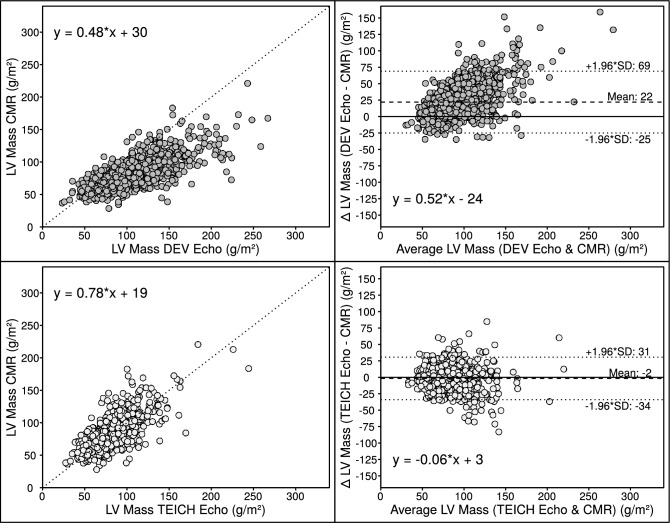
Correlation plots (left panels) with line of identity (dotted) and Bland–Altman plots (right panels) with mean bias (dashed) and limits of agreement (dotted horizontals) for the DEV and TEICH method with CMR as the reference imaging in the derivation cohort (n = 1276).

The main Echo and CMR characteristics of LV measurements are summarized in Table 2. The LV myocardial mass calculated by the Echo DEV and TEICH methods correlated similarly with volumetric mass measurements derived from CMR imaging in the derivation and validation cohort (ICC = 0.66 and 0.84; ICC = 0.51 and 0.75; *p* < 0.001 for all). However, the DEV method exhibited a proportional bias with increasing mass for the derivation and validation cohort. TEICH exhibited virtually no bias vs. CMR in both cohorts (mean bias: − 2 and + 3 g/m^2^). Results of the correlation and the Bland–Altman analyses of the derivation cohort are shown in Fig. 3, and the results of the validation cohort are shown in the Supplemental data (Fig. S3).

**Table 2:** Echocardiographic and CMR parameters of the study participants.

LV parameters	Derivation cohort (n = 1276)	Validation cohort (n = 226)
Echo		
EDD, cm	4.9 (4.4;5.4)	5.6 (4.9;6.3)
IVS, cm	1.0 (0.8;1.3)	1.1 (0.9;1.4)
PW, cm	1.0 (0.8;1.1)	0.8 (0.7;1.1)
Mean Wall Thickness, cm	1.0 (0.8;1.2)	1.0 (0.8;1.3)
Mass, g/m^2^, DEV formula	94 (74;119)	105 (90;124)
Mass, g/m^2^, TEICH formula	73 ( 61;88)	82 (73;93)
CMR		
Mass, g/m^2^	73 (61;88)	81 (73;92)
LGE presence, n (%)	401 (31)	165 (73)

Data are presented as median with interquartile ranges (25th percentile;75% percentile).

CMR, cardiac magnetic resonance; DEV, Devereux; ECHO, echocardiography; EDD, end-diastolic diameter; IVS, interventricular septum; LV, left ventricular; LGE, late gadolinium enhancement; PW, posterior wall; TEICH, Teichholz.

### Correlates of measurement bias of left ventricular mass in echocardiography

**Figure 4:**
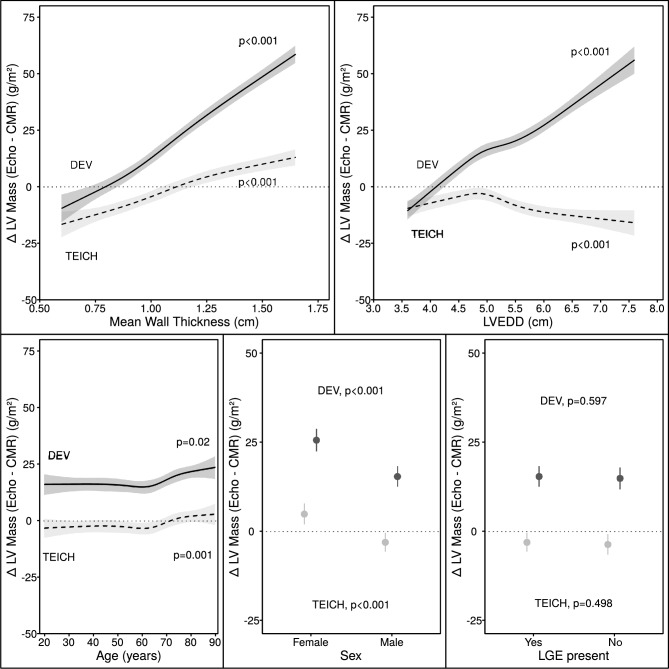
Multivariable regression fits of the inter-method bias of LV mass index (g/m^2^) as the dependent variable for the DEV (solid) and TEICH (dashed) method vs. CMR imaging as the reference method for mean wall thickness, LV EDD, age and sex as the independent variables in the derivation cohort (n = 1276).

Multivariable analysis in the derivation cohort confirmed that primarily LV EDD and secondary wall thickness were the main sources of discrepancy between the TEICH and DEV methods for LV mass measurement as shown in the Supplemental data in Fig. S4. Further regression analyses were conducted to identify the specific factors contributing to measurement bias, including the presence of LGE, age and sex (Table 3 and Table S2 in the Supplemental data). Results showed that all independent features were less strongly associated with measurement bias in the TEICH method compared to the DEV method. The TEICH method showed an attenuated and inverse u-shaped association with LV diameter (Fig. 4, Fig. S5 and Table S2 in the Supplemental data). Hence, TEICH underestimated mass when compared to the CMR analyses in patients comprising a dilated left ventricle, while the DEV method overestimated them. Although LGE was not statistically significant in multivariable regression, DEV-calculated LV mass showed medians of 108 g/m^2^ (86;134) in patients with LGE and 87 g/m^2^ (71;109) without, while TEICH-calculated mass showed medians of 81 g/m^2^ (69;96) with LGE and 70 g/m^2^ (59;82) without. The bias to CMR mass with DEV was 24 g/m^2^ (10;42) with LGE and 16 g/m^2^ (5;30) without LGE, whereas TEICH demonstrated minimal bias with LGE (0 g/m^2^, − 10; 9) and a slight negative bias without LGE (− 3 g/m^2^, − 11; 7). Similar results were found for patients with known or suspected coronary artery disease (Fig. S6 in the Supplemental data).

**Table 3:** Multivariable regression analyses of measurement bias in LV mass index (g/m^2^) as response variable from ECHO versus CMR imaging for DEV and TEICH on derivation cohort (n = 1276).

Parameters	DEV-bias model		TEICH-bias model	
	Effect (SE)	*P*-value	Effect (SE)	*P*-value
LV mean wall thickness, cm, 0.8–1.3	34.8 (1.3)	< 0.001	16.5 (1.2)	< 0.001
LV EDD, cm, 4.5–5.9	17.4 (1.3)	< 0.001	− 6.4 (1.2)	< 0.001
LGE, yes versus no	− 0.5 (0.9)	0.597	− 0.6 (0.9)	0.498
Age, years, 51–75	4.7 (1.3)	< 0.001	3.8 (1.2)	< 0.001
Sex, female versus male	10.2 (1.0)	< 0.001	7.9 (0.94)	< 0.001
Model fit				
Adjusted R^2^ (Predictive R^2^)	0.59 (0.60)	< 0.001	0.25 (0.26)	< 0.001

DEV, Devereux; CMR, cardiac magnetic resonance; ECHO, echocardiography; EDD, end-diastolic; LV, left ventricular; LGE, late gadolinium enhancement; SE, standard error of effect; TEICH, Teichholz; mean wall thickness, average thickness of interventricular septum and posterior wall.

R^2^ denotes the regression coefficient and *P*-value represents the significance value of the F-statistic from analysis of variance test for each parameters or the whole model.

### Impact of tier classification on myocardial mass estimation by echocardiography

**Figure 5:**
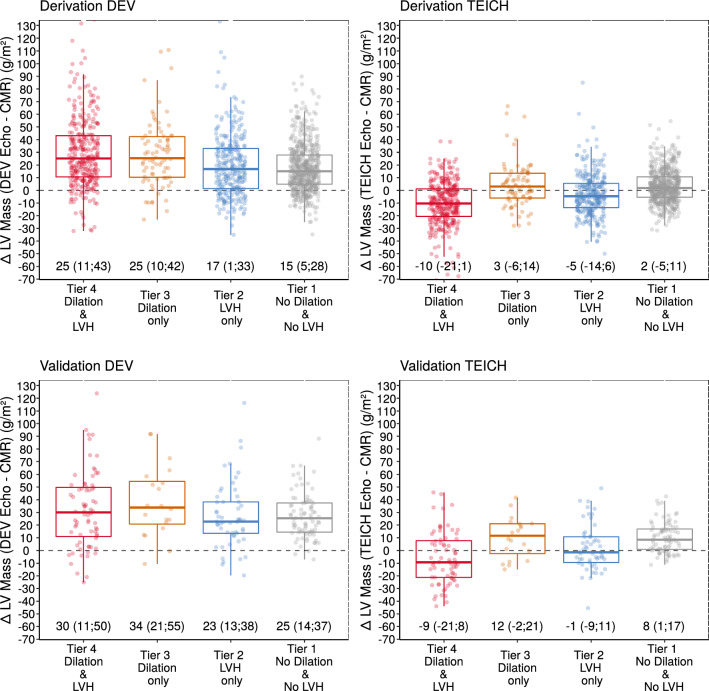
Subgroup analyses represented by boxplots showing the measurement differences of LV mass (Δ) for the derivation cohort (n = 1276, top row) and the validation cohort (n = 226, bottom row), respectively, as well as for the DEV (left panels) and the TEICH method (right panels), and illustrating the systematic overestimation of LV mass with the DEV equation compared to CMR as the reference imaging method for each tier of LV geometry.

The tier criteria of LV hypertrophy and dilation based on reference values of CMR imaging are shown in Table 4. In line with the mathematical calculations and the regression results on measurement bias (Figs. 2 and 4), the DEV method by ECHO systematically overestimated LV mass compared with the CMR technique in all 4 tiers for the derivation as well for the validation cohort (Fig. 5). The TEICH formula showed an underestimation of LV myocardial mass in patients with severe LV dilatation and eccentric hypertrophy (i.e., tier 4), but a smaller overestimation in tier 3 compared to the DEV method (derivation cohort: 3 vs. 25 g/m^2^; validation cohort: 12 vs. 34 g/m^2^, *p* < 0.001 for all).

**Table 4:** Tier classification of left ventricular geometry.

	Derivation cohort (n = 1276)	Validation cohort (n = 226)
Tier 1: no LV dilation & no LVH, n (%)	575 (45)	81 (36)
Tier 2: LVH only, n (%)	293 (23)	53 (23)
Tier 3: LV dilation only, n (%)	82 (6)	20 (9)
Tier 4: LV dilation & LVH, n (%)	326 (26)	72 (32)

Tier classification criteria based on CMR imaging reference values according to Kawel-Boehm and colleagues ^15^ as described in Supplemental data Table S1.

CMR, cardiac magnetic resonance; LV, left ventricular; LVH, left ventricular hypertrophy.

#### Observer variability

The intra- and inter-observer ICC values for measurements obtained from both Echo and CMR are presented in Table 5. For CMR measurements, both intra- and inter-observer ICCs were excellent. Conversely, for Echo measurements, only intra-observer ICCs exhibited good to excellent values.

**Table 5:** Observer variability of CMR and Echo measurements (n = 35).

	Intra-observer ICC (95% confidence interval)	Inter-observer ICC (95% confidence interval)	Inter-observer CV (%)
CMR:			
LV mass	0.98 (0.96–0.99)	0.97 (0.96–0.99)	3.7
LV EDV	0.99 (0.99–0.99)	0.99 (0.98–0.99)	3.0
Echo:			
EDD, cm	0.91 (0.87–0.94)	0.83 (0.69–0.90)	8.5
IVS, cm	0.85 (0.80–0.90)	0.77 (0.61–0.87)	15.8
PW, cm	0.75 (0.68–0.84)	0.61 (0.15–0.81)	15.1
Mass: DEV formula	0.82 (0.72–0.91)	0.69 (0.48–0.83)	16.3
Mass: TEICH formula	0.82 (0.71–0.91)	0.69 (0.49–0.83)	14.0

ICC, intraclass correlation coefficient; CV, coefficient of variation; CMR, cardiac magnetic resonance; LV, left ventricular; EDV, end-diastolic volume; DEV, Devereux; ECHO, echocardiography; EDD, end-diastolic diameter; IVS, interventricular septum; PW, posterior wall; TEICH, Teichholz.

## Discussion

The main findings of the study are: 1) Mathematical computation of the DEV equation postulated a systematic overestimation of LV mass in dilated left ventricles, which increases with further incremental wall thickening. 2) Using these findings, we were able to elucidate that the LV diameter is the main source of disproportionate error in the DEV method and 3) the TEICH equation incorporates a flexible correction factor that enables closer agreement with the gold standard CMR than the DEV method in 4) almost all LV morphology, except in cases with the most severe LV geometrical aberrations. 5) We assessed the reproducibility of the TEICH method for different clinical entities on a timely independent cohort.

Recent studies questioned the accuracy of the DEV method in patients with concentric LV hypertrophy^7,9,13^. Both methods (Fig. 1), DEV and TEICH, rely on geometric assumptions of the left ventricle as a prolate ellipsoid based on the fundamental CUBE equation refined by Dodge and colleagues^11,12^. However, we demonstrated that the TEICH method is less affected by these geometric assumptions than the DEV method on a large cohort (Figs. 2 and 3). The TEICH method seems to flexibly follow pattern changes of adverse LV remodeling, adhering to the measurements of the gold standard CMR imaging as shown in our study (Fig. 5). This is mainly due to the modifications performed by Teichholz and colleagues in the aforementioned CUBE equation^10^. TEICH and DEV incorporate exactly the same input values of LV cross section for mass calculation (i.e., IVS, EDD and PW). In a mathematical sense, the only modification of the TEICH equation is the correction parameter, which aims to minimize the error in the fundamental CUBE formulation if the geometry of the LV cavity is distorted. Indeed, the inherent assumptions of the DEV technique assume a fixed (i.e. symmetric) geometry of the ventricle, resulting in an overestimation of LV mass, especially with the progressive ventricular dilation typical for ischemic heart disease^8^. In line with these findings, the TEICH method yielded lower measurement variation across the 4-tier classification of LV morphology, and TEICH did not exhibit the same proportional measurement bias as the DEV method (Figs. 3 and 5). With the help of mathematical modeling (Fig. 2), we demonstrated that increasing LV size is the main source of LV mass discrepancy between the DEV and TEICH (Fig. 4). Although we used B-mode measurements of the LV cross section, our findings are consistent with a previous study on selected patients with LV hypertrophy^7^. Nevertheless, the TEICH method also encounters its limits in patients with progressive LV dilation and concomitant eccentric hypertrophy (i.e. tier 4), but the differences between TEICH and CMR imaging were less pronounced than those between DEV and CMR (Fig. 5). Salgo and colleagues^25^ conducted a curvature analysis in non-ischemic and ischemic cardiomyopathies, revealing a range of LV shapes that underscore diverse LV symmetries. These configurations are suggestive of the nature and extent of myocardial injury, and reciprocally, they exert an influence on prognosis^26^. In the absence of additional LV geometric and myocardial injury data, we can only speculate about the potential influence of diverse remodeling on the performance of the DEV and TEICH methods, as showcased in tier 4 of our study. This highlights the imperative for further research, encompassing comprehensive LV shape analysis, detailed LGE extent and localization data, and advanced CMR parameters like extracellular volume, to gain deeper insights. However, in patients with isolated LV dilation, LV hypertrophy or normal LV geometry the TEICH calculations show good agreement with CMR imaging. Certainly, the calculation of LV mass with one-dimensional measurements is based on geometric assumptions of the fundamental CUBE method and is prone to technical errors and varies highly dependent on the level of measurement especially in distorted left ventricles^17,27^. Indeed, our study revealed a notable disparity in measurement inconsistency, notably evident in posterior LV wall thickness, followed by the septum (Table 5). Strikingly, the measurement of LV end-diastolic diameters emerged with the strongest intra- and inter-observer agreement. This finding offers a potential explanation for the convergence in reproducibility characteristics observed between the TEICH and DEV methods. Our study underscores the central role of end-diastolic diameter as a primary contributor to measurement bias, a factor of utmost importance, as exemplified in Fig. 4. Therefore, Kristensen and colleagues proposed an alternative method for mass calculation in two-dimensional Echo: summing up the mean LV wall thickness measured in the parasternal short axis with the biplane end-diastolic LV volume measured in the apical long axis^28^. Although this new method provides more precise results than the DEV method, it requires a more elaborate measurement technique than the TEICH method.

Given that several outcome studies have demonstrated that LV mass measured by DEV provides prognostic information, one may question the rationale for assessing the accuracy of an already known method like TEICH. However, as our comprehensive analysis underscores (Figs. 4 and 5), the DEV method consistently overestimates LV mass relative to CMR measurements, irrespective of LV geometry. This is particularly notable in patients with thickened and dilated LVs—indicative of a poorer prognosis—suggests that the usage of the DEV method in prognostication might surpass that of the TEICH formula. Nonetheless, prospective clinical trials are warranted, especially to ascertain potential false positive results attributable to the DEV formula. In addition, considering the established prognostic value of LV mass measured by one-dimensional Echo, and the known limitations of the widely used DEV formula in certain patient cohorts, the need for evaluating the accuracy of alternative LV mass calculation methods like TEICH becomes evident. Firstly, the use of the TEICH method may represent a step towards containing the conglomeration of different normal reference ranges and achieving a unifying level, with CMR imaging as the non-invasive gold standard. Secondly, prognostic information is surely of utmost importance; however, it is vital that a method shows high accuracy, as we have demonstrated with the TEICH method. At last, external clinical validation of our finding is still pending, but the use of the TEICH formula instead of the DEV formula should be considered when performing feasible one-dimensional, linear measurements for LV mass calculation in Echo. We believe that our findings may spur further research.

### Limitations

We acknowledge several limitations of this single-center study. Men were over-represented in both the derivation and the validation cohort. However, our study included a large number of patients with a wide range of LV morphology and various entities of cardiac disease that are commonly encountered in clinical practice. LV mass calculations using DEV and TEICH methods were based on B-mode measurements, which may limit comparability with previous studies using M-mode measurements. However, we used B-mode measurements for both methods and clearly demonstrated the higher accuracy of the TEICH equation. While the presence of LV myocardial LGE could potentially affect the DEV method more adversely than the TEICH method, it is important to note that our study did not evaluate a more nuanced spectrum of LGE characteristics. Finally, it is important to note that our validation cohort consisted of a restricted number of patients. Therefore, further research is necessary to fully assess the utility of the TEICH method in clinical context.

## Conclusion

Our analyses show that the DEV method based on Echo B-mode measurements systematically overestimates LV mass. In contrast, the TEICH method provides measurement results comparable to those obtained from CMR imaging. The advantage of the TEICH method is that it takes into account the change in LV geometry with increasing LV, and thus reflects the different pattern of LV remodeling.

### Supplementary Information

Supplementary Information.

## Data Availability

The data that support the findings of this study are available on request from the corresponding author, K.M.S. The data are not publicly available due to restrictions of the federal law.
