# Author Correction: Accuracy of Devereux and Teichholz formulas for left ventricular mass calculation in different geometric patterns: comparison with cardiac magnetic resonance imaging

**DOI:** 10.1038/s41598-023-43066-1

**Published:** 2023-09-22

**Authors:** Krunoslav Michael Sveric, Barış Cansız, Anna Winkler, Stefan Ulbrich, Georg Ende, Felix Heidrich, Michael Kaliske, Axel Linke, Stefanie Jellinghaus

**Affiliations:** 1 Department for Internal Medicine and Cardiology, Herzzentrum Dresden, Technische Universität Dresden, Fetscherstr. 76, 01307 Dresden, Germany; 2 Institute for Structural Analysis, Technische Universität Dresden, 01062 Dresden, Germany

Correction to: *Scientific Reports* 10.1038/s41598-023-41020-9, published online 28 August 2023

The original version of this Article contained an error in the title of the paper, where the term “Devereux” was incorrectly given as “Devereaux”.

In addition, in the Abstract,

“We aimed to compare the accuracy of the Devereaux formula (DEV) and the Teichholz formula (TEICH) in calculating LV myocardial mass in Echo using cardiac magnetic resonance (CMR) as the reference method.”

now reads:

“We aimed to compare the accuracy of the Devereux formula (DEV) and the Teichholz formula (TEICH) in calculating LV myocardial mass in Echo using cardiac magnetic resonance (CMR) as the reference method.”

The original Article and accompanying Supplementary Information file have been corrected.

