# Supplementary material for: Accuracy of Devereux and Teichholz formulas for left ventricular mass calculation in different geometric patterns: comparison with cardiac magnetic resonance imaging
Source: Sci Rep. 2023 Aug 28;13:14089. doi: 10.1038/s41598-023-41020-9 (PMC10462733; doi:10.1038/s41598-023-41020-9)
Supplement: Supplementary file 1 — Supplementary Information. [file 41598_2023_41020_MOESM1_ESM.docx]

Supplemental Data

**Accuracy of Devereux and Teichholz formulas for left ventricular mass calculation in different geometric patterns: comparison with cardiac magnetic resonance imaging.**

*Krunoslav Michael Sveric^a^, MD; Barış Cansız^b^, PhD; Anna Winkler^a^, MD; Stefan Ulbrich^a^, MD; Georg Ende^a^,MD; Felix Heidrich^a^, MD; Michael Kaliske^b^, PhD; Axel Linke^a^, MD; Stefanie Jellinghaus^a^, MD;

^a^ Department for Internal Medicine and Cardiology, Herzzentrum Dresden, Technische Universität Dresden, Fetscherstr. 76, 01307 Dresden, Germany

^b^ Institute for Structural Analysis, Technische Universität Dresden, 01062 Dresden, Germany

**Methods**

**Supplemental Tables and Figures**

| **Table S1**: Tier classification criteria of LV geometry based on CMR imaging (ages 15 – 83 years) | | |
| --- | --- | --- |
| Sex | Female | Male |
| LV myocardial mass index, g/m² | >59 | >75 |
| LV end-diastolic volume index, ml/m² | >93 | >107 |
| Reference values are based on Table 5 from Kawel-Boehm N., Hetzel SJ., Ambale-Venkatesh B., et al. Reference ranges (“normal values”) for cardiovascular magnetic resonance (CMR) in adults and children: 2020 update. vol. 22. BioMed Central; 2020 – Reference #16 of the main manuscript. Upper limits have been calculated in the review from + 2 times standard deviation from the mean. | | |

**Fig. S1:** Flow chart depicting the screening procedure of patients for the derivation cohort.

**Results**

**Supplemental Tables and Figures**

**
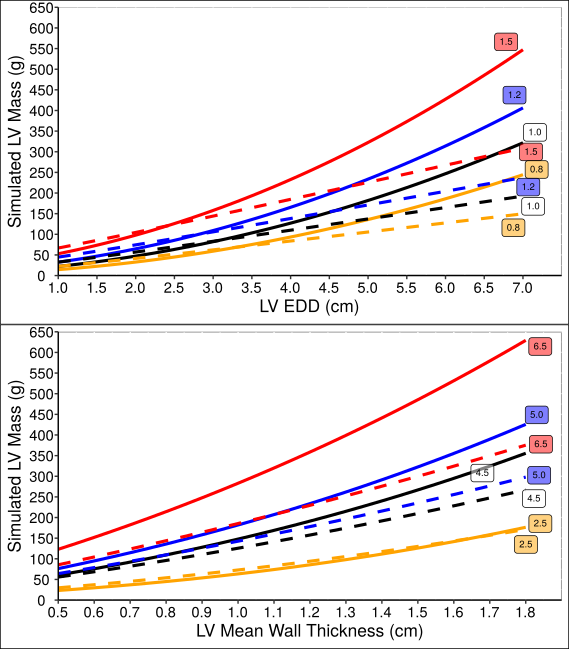
**

**Fig. S2:** Comparison of univariable mathematical calculations for the DEV (solid) and the TEICH (dashed) method. Upper panel: Incremental increase of EDD ranging from very small 1 cm to very dilated 7 cm, while mean wall thickness (i.e. mean of IVS and PW) was hold constant for following levels: 0.8, 1.0, 1.2 and 1,5 cm.

Lower panel: The mean wall thickness was incrementally increased ranging from very thin 0.5 cm to very thickened 1.8 cm, while EDD was hold constant for following levels: 2.5, 4.5, 5.0 and 6.5 cm.

**Fig. S3:** Correlation plots (left panels) with line of identity (dotted) and Bland-Altman plots (right panels) with mean bias (dashed) and limits of agreement (dotted horizontals) for the DEV and TEICH method with CMR as the reference imaging in the validation cohort (n = 226). The linear equation represents the fit of data between the methods, but also denotes the proportional bias of the DEV Echo method that is not observed for TEICH.

**
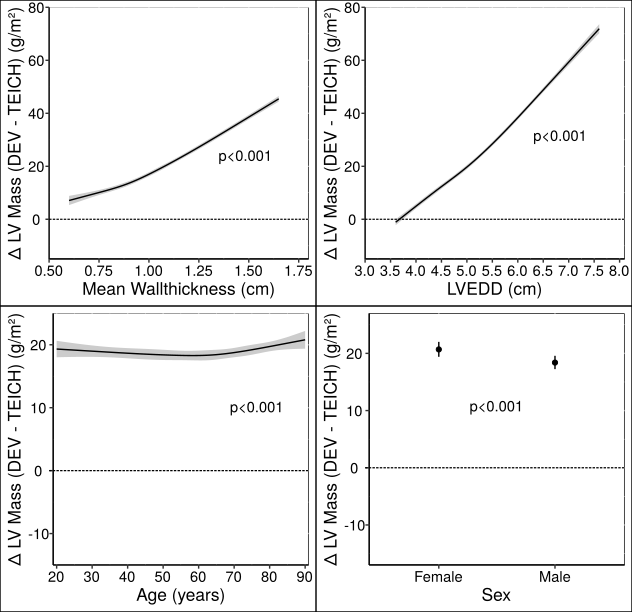
**

**Figure S4:** Regression analysis of measurement bias between DEV and TEICH method on multivariable regression fits of the inter-method for mean wall thickness, LV EDD, age and sex as the independent variables in derivation cohort (n = 1,276).

| **Table S2**: Multivariable regression analyses of measurement bias in LV mass index (g) as response variable from ECHO versus CMR imaging for DEV and TEICH on derivation cohort (n = 1,276) | | | | |
| --- | --- | --- | --- | --- |
|  | ~~CUBE~~ DEV-bias model | | TEICH-bias model | |
| Parameters | Effect (SE) | P-value | Effect (SE) | P-value |
| LV mean wall thickness, cm, 0.8 to 1.3 | 73.2 (2.6) | <0.001 | 32.8 (2.6) | <0.001 |
| LV EDD, cm, 4.5 to 5.9 | 37.7 (2.5) | <0.001 | -13.8 (2.4) | <0.001 |
| LGE, yes vs. no | -1.8 (1.9) | 0.346 | -1.2 (1.8) | 0.506 |
| Age, years, 51 to 75 | 6.9 (2.5) | <0.001 | 8.1 (2.4) | <0.001 |
| Sex, female vs male | 17.0 (2.0) | <0.001 | 15.5 (1.9) | <0.001 |
| Model fit |  |  |  |  |
| Adjusted R² (Predictive R²) | 0.61 (0.60) | <0.001 | 0.26 (0.25) | <0.001 |
| DEV = Devereux; CMR = cardiac magnetic resonance; ECHO = echocardiography; EDD = end-diastolic; LV = left ventricular; LGE = late gadolinium enhancement; SE = standard error of effect; TEICH = Teichholz; mean wall thickness = average thickness of interventricular septum and posterior wall.  R² denotes the regression coefficient and P-value represents the significance value of the F-statistic from analysis of variance test for each parameters or the whole model. | | | | |

**Figure S5:** Regression analysis of measurement bias (LV mass in g) between DEV and TEICH method on multivariable regression fits of the inter-method for mean wall thickness, LV EDD, LGE, age and sex as the independent variables in derivation cohort (n = 1,276).


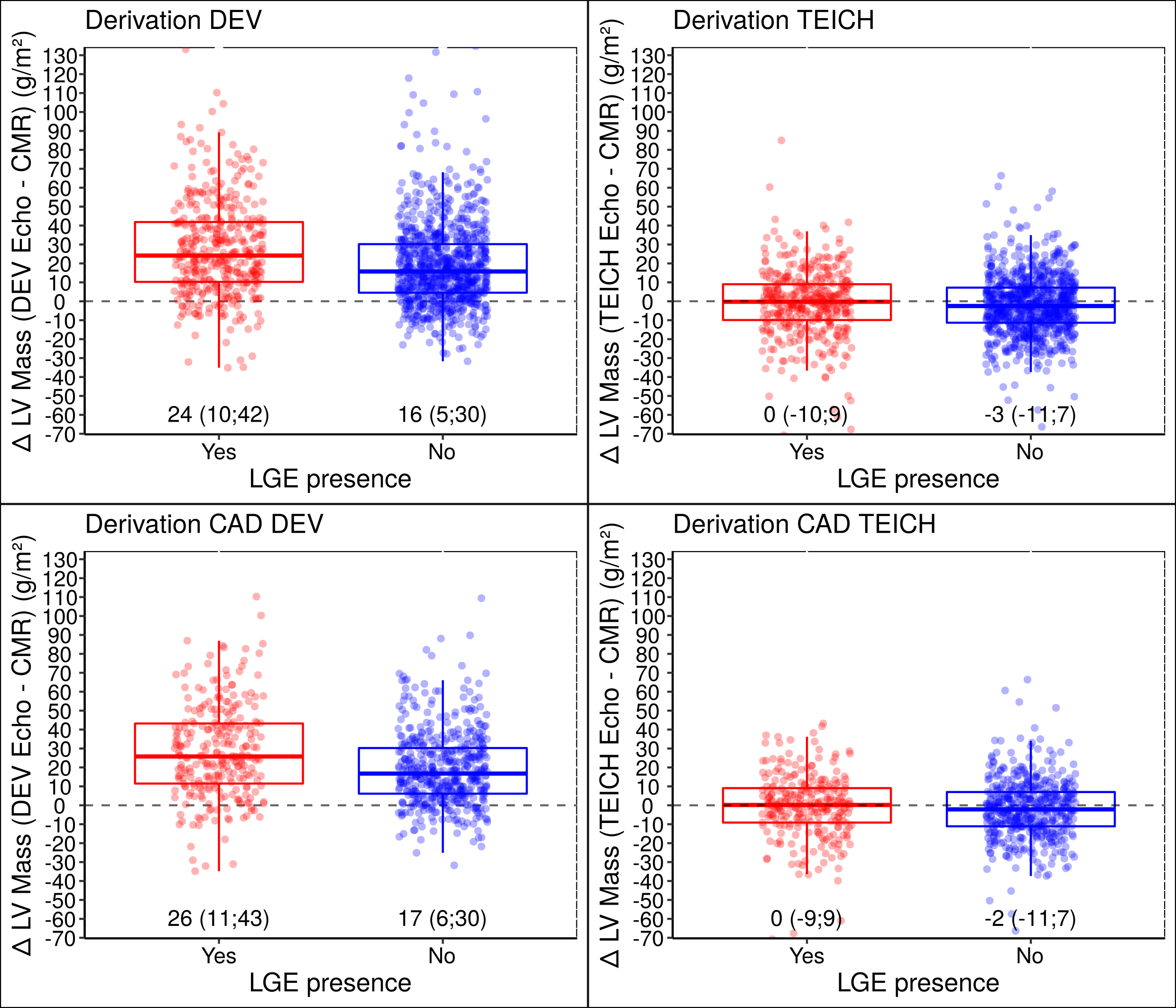


**Figure S6:** Subgroup analyses represented by boxplots showing the measurement differences of LV mass (Δ) for the entire derivation cohort (n = 1,276) and patients with existing or suspected CAD (n = 755, bottom row), respectively, as well as for the DEV (left panels) and the TEICH method (right panels), and illustrating the systematic overestimation of LV mass with the DEV equation compared to CMR as the reference imaging method for the presence of LV LGE.
